# Characterizing the 2020 summer floods in South China and effects on croplands

**DOI:** 10.1016/j.isci.2023.107096

**Published:** 2023-06-12

**Authors:** Xi Chen, Jinwei Dong, Lin Huang, Lajiao Chen, Zhichao Li, Nanshan You, Mrinal Singha, Fulu Tao

**Affiliations:** 1 Key Laboratory of Land Surface Pattern and Simulation, Institute of Geographic Sciences and Natural Resources Research, Chinese Academy of Sciences, Beijing 100101, China; 2 Aerospace Information Research Institute, Chinese Academy of Sciences, Beijing 100094, China; 3 College of Resources and Environment, University of Chinese Academy of Sciences, Beijing 100049, China; 4 Natural Resources Institute Finland (Luke), 00790 Helsinki, Finland

**Keywords:** Atmospheric science, Meteorology, Global change, Agricultural science

## Abstract

Floods occur more frequently in the context of climate change; however, flood monitoring capacity has not been well established. Here, we used a synergic mapping framework to characterize summer floods in the middle and lower reaches of the Yangtze River Plain and the effects on croplands in 2020, from both flood extent and intensity perspectives. We found that the total flood extent was 4936 km^2^ from July to August, and for flood intensity, 1658, 1382, and 1896 km^2^ of areas experienced triple, double, and single floods. A total of 2282 km^2^ croplands (46% of the flooded area) were inundated mainly from Poyang and Dongting Lake Basins, containing a high ratio of moderate damage croplands (47%). The newly increased flooding extent in 2020 was 29% larger than the maximum ever-flooded extent in 2015–2019. This study is expected to provide a reference for rapid regional flood disaster assessment and serving mitigation.

## Highlights

• A comprehensive assessment of extent and intensity of 2020 summer floods in MLYP
• Mapping floods by integrating the CDAT-based and NDFI-based methods
• The 2020 newly increased flooding extent was 29% larger than that of 2015–2019
• 2282 km^2^ croplands were inundated during the 2020 floods in MLYP

## Graphical abstract

**Figure:**
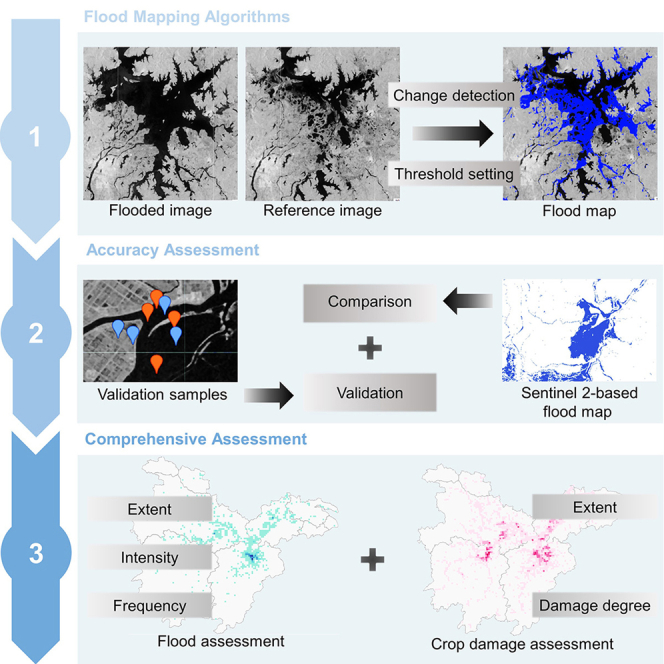

## Introduction

Floods are one of the most frequent and severe climate-related disasters, threatening human livelihoods and properties.^1^^,^^2^ Agricultural production could be seriously damaged by floods. According to the United Nations (UN), 70% of the world’s rain-fed agriculture and 1.3 billion people who depend on cropland face great threats from the changes in rainfall patterns.^3^ In the context of climate change, due to an increase in storm frequency and intensity with respect to the previous decades, there is a rising trend of floods worldwide.^4^^,^^5^^,^^6^ Timely and accurate information about floods (e.g., the spatial extent and intensity of floods) is of particular importance as it provides necessary information for defining the strategies for disaster prevention and post-disaster recovery in the agricultural sector, and then benefits food security.

Although flood identification based on traditional river gauge data can predict the numerical variation of flood areas on a national scale, it is unable to provide the spatial pattern of flooded areas.^4^^,^^7^ Studies on flood mapping that provides spatial distribution information could be generally divided into post-event, real-time, and near real-time mapping in terms of the acquisition time of hydrological data used. The post-event flood mapping utilizes multi-temporal data (e.g., gauge-station data, run-off data, aerial and satellite imagery, and documented historical flood maps) and various hydraulic flood models^8^ after the flooding events for long-term damage assessment after severe floods.^9^ These models are only suitable for small or medium-scale flood mapping and short river stretches with a few tributaries, such as HEC-RAS^10^ and LISFLOOD-FP.^11^ For example, Afghanistan Flood Hazard Map (AFG-FHM)^8^ has been proposed to extract the nationwide flood extent with inadequate hydrological data; however, it cannot provide the spatial patterns of flood dynamics. In addition, real-time mapping utilizes the data from water gauge sensors and timely official flooding reports to produce a flood probability map; however, it is difficult to extract timely flood information at a large scale due to the limitation of real-time hydrological observation data.^12^ In contrast, the near real-time flood map could achieve flood monitoring shortly after the flooding event, providing a feasible approach for the timely and accurate large-scale flood damage assessment.

Remote sensing has been increasingly used in near real-time flood monitoring in recent decades. Because the flooding is often accompanied by precipitation and cloud cover, active satellite-based sensors such as synthetic aperture radar (SAR) are more suitable for near real-time flood monitoring than optical sensors, as they allow for earth observation under all weather conditions.^13^ Efforts have been made to extract flood information based on SAR images,^14^^,^^15^^,^^16^^,^^17^^,^^18^^,^^19^ which could be clustered into three categories: (1) single-image-based flood mapping,^15^^,^^20^ (2) change-detection-based flood mapping,^21^^,^^22^ and (3) multi-temporal flood mapping.^16^^,^^23^ Compared with the single image-based methods, change-detection-based approaches use flood-related backscatter reduction to distinguish instantaneous floods from permanent water bodies. As permanent water bodies have not changed remarkably during this period, the approaches enable overcoming the shortcomings of backscattering heterogeneity caused by the confusion of dry and submerged vegetation.^15^^,^^17^ The accuracy of the method highly depends on the selection of non-flood reference images.^6^^,^^24^ However, change-detection-based methods only using a single image in the flood period could not depict entire large-scale flood mapping. Mapping with multi-temporal data could realize wall-to-wall flood mapping and provide a better understanding of the seasonal behavior of different land cover types.^14^ Time-series analyses rely on high revisit rate and availability of SAR data with complex processing. The Google Earth Engine (GEE) platform constantly updates the Sentinel-1 (S1) SAR data and hosts the algorithms of time series data processing,^25^ which makes it possible to process near real-time flooding monitoring.^26^ In this case, a combination of the change detection and thresholding(CDAT)^21^ and the Normalized Difference Flood Index (^18^ algorithms with multi-temporal data have initially proven to be reliable in accurate and effective flood mapping.^27^

A comprehensive analysis of extent, intensity, and frequency of floods can contribute to the precise management and prevention especially the cropland damage in severe flood-affected areas. Previous studies mainly focused on flood extent in a single period^14^^,^^28^^,^^29^ or multi-year flood frequency^27^^,^^30^ and hardly considered flood intensity that reflects times an inundated area at different stages of a flood event. In addition, most previous studies focus on local-scale floods, and large-scale flood events have not been well investigated.

In the summer of 2020, South China suffered severely from catastrophic floods. Large-scale floods mainly occurred in the middle and lower reaches of the Yangtze River Plain (MLYP). In July 2020, affected by the continuous heavy rainfall, the river level of the MLYP and the main lakes continued to exceed the warning level. The water level of Taihu Lake exceeded the maximum level, and the water level of some stations in Poyang Lake reached the highest historical record. In August 2020, the Minjiang River and Jialing River in the upper reaches of the Yangtze River experienced another huge flood. According to the Ministry of Emergency Management (MEM) of the People’s Republic of China, the floods of MLYP in the summer of 2020 influenced 34.2 million people in 11 provinces, with a direct economic loss of 132.2 billion yuan. Under such circumstances, near real-time monitoring of floods and flood dynamics in the Yangtze River basin is absolutely and urgently needed.^31^ However, existing studies focused on the parts of MLYP,^32^^,^^33^^,^^34^ such as Poyang Lake and Chao Lake Basin, whereas few studies concerned flood monitoring and flood severity assessment of whole MLYP.

In this context, this paper aims to combine the change-detection-based approaches and multi-temporal data to realize rapid and accurate flood monitoring in MLYP, provide a comprehensive assessment of the flood severity, and analyze the flood impact on croplands. Specifically, based on S1 SAR multi-temporal data derived from GEE platform, we use the synergy of the two change monitoring methods (i.e., CDAT and NDFI) to obtain flood maps of the MLYP from 2015 to 2020 and cropland inundation map in 2020, aiming to answer the following scientific questions: (1) is the synergy of CDAT and NDFI algorithms suitable for mapping flood of the MLYP? (2) compared with previous years, what is the specific manifestation of flood severity in 2020? and (3) how does the flood disaster impact croplands in 2020?

## Results

### Accuracy of flood maps

**Figure 1:**
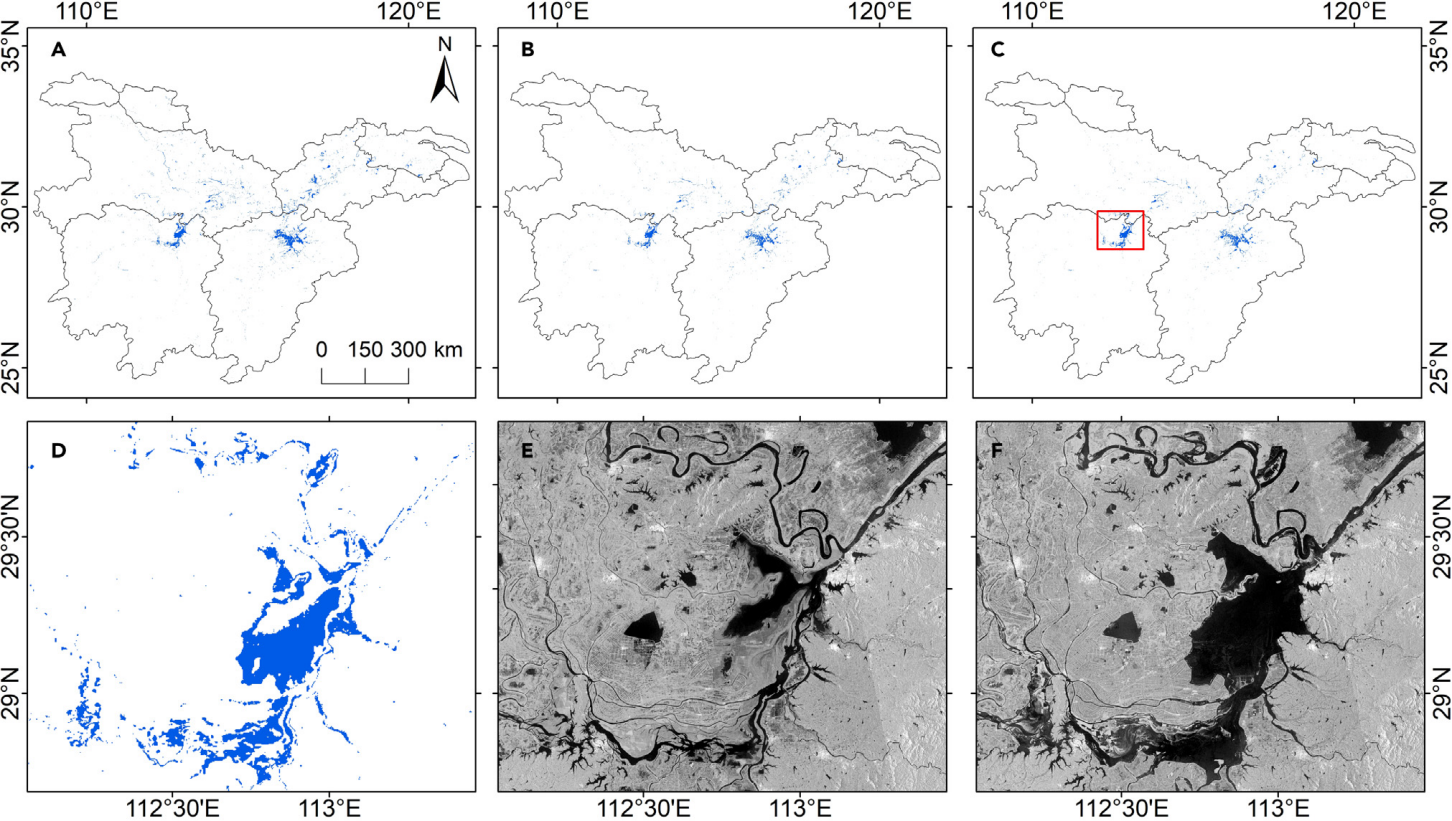
Flooded areas derived from two algorithms and S1 images from July to August 2020 (A) Using CDAT algorithm; (B) using NDFI algorithm; (C) consistency map (flooded areas common for both the CDAT and NDFI algorithm); (D) zoom area from the consistency map showing part of Dongting Lake Plain; (E) reference image in the zoom area; (F) flood image in the zoom area.

The proposed method permits to build an accurate, consistent flood map with an overall accuracy (OA) of 96.5% and an F-score value of 0.93 (Table 1). The user’s accuracy (UA) and producer’s accuracy (PA) of the flooded class were 99.48% and 92.01%, indicating that the result had low omission (truly flooded pixels not identified as such) and commission (pixels identified as flooded area that were non-flooded) errors. We also conducted a visual comparison between flood maps and reference images to assess the ability of flood maps to discriminate between flood and permanent water. Figure 1C shows the consistency flood map by integrating NDFI (Figure 1A) and CDAT (Figure 1B) algorithms. The zoom area gave reasonable flooded areas compared to the reference image in part of Dongting Lake Plain (Figures 1D–1F).

**Table 1:** Confusion matrix for accuracy assessment based on the validation samples from June to August 2020

Class	Flooded	Non-flooded	User’s accuracy	Producer’s accuracy	Overall accuracy	F1-score
Flooded	380	2	99.48	92.01	96.50	0.93
Non-flooded	33	585	94.66	99.66

**Figure 2:**
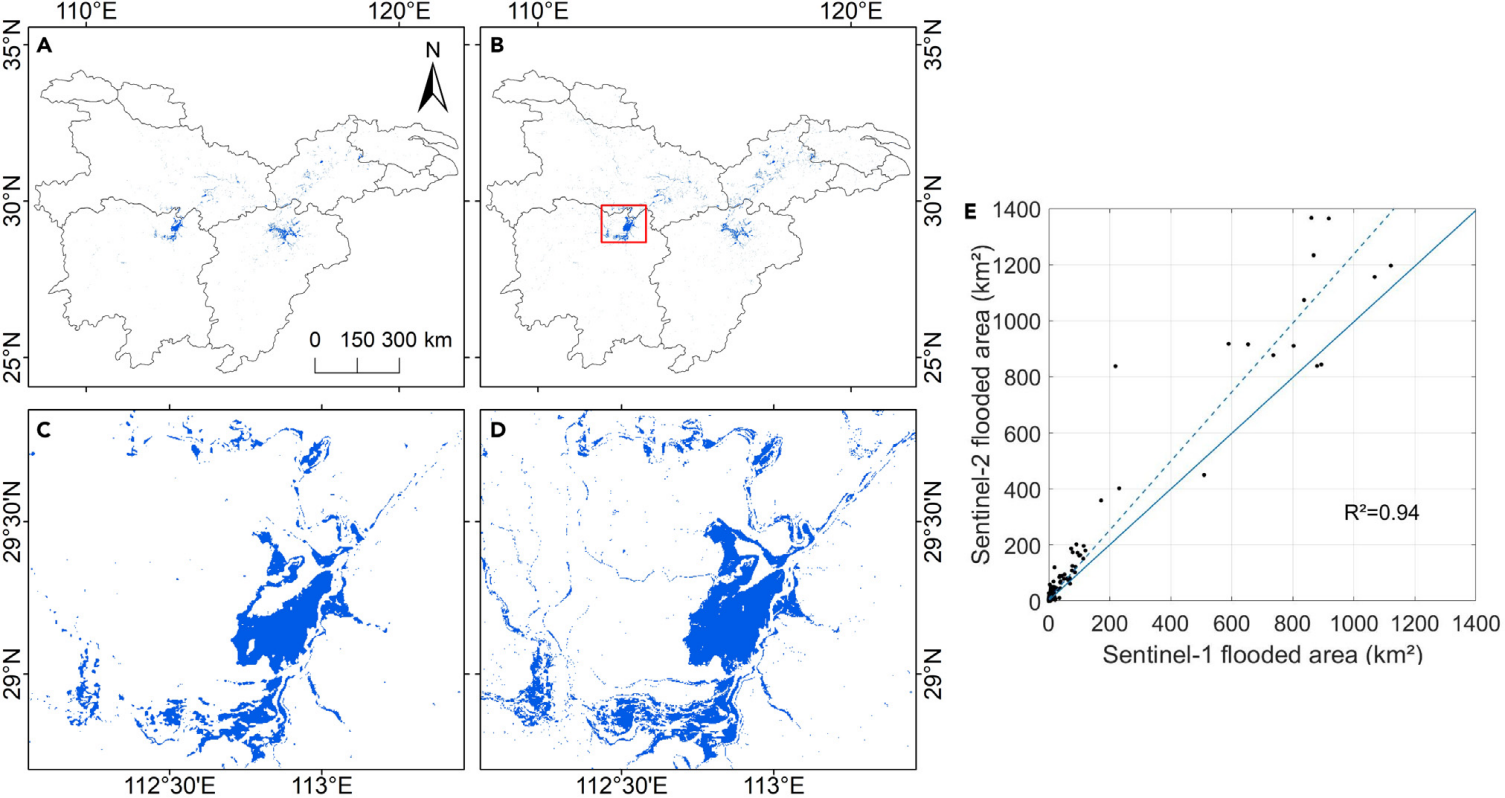
Comparison between S1-based and S2-based flood maps (A) S1-based flood map; (B) S2-based flood map; zoom area from the S1-based (C) and S2-based (D) flood maps showing part of Dongting Lake Plain; (E) scatterplot for comparison between S1-based and S2-based flood area at the county level in 2020.

Furthermore, the intercomparison of two maps is spatially consistent in flooded areas (Figures 2A and 2B), and the Sentinel-2 (S2) -based flood map extracted additional flood areas on the Dongting Lake Plain (Figures 2C and 2D). Figure 2E shows the flooded area summed at the county level between the S1-based and S2-based flood maps. Most of the scatters are distributed along the 1:1 line with a coefficient of determination (R^2^) of 0.94, indicating that the two maps have a relatively good correlation. However, the S2-based flood map overestimated floods caused by the following reasons. Firstly, the spatial resolution of 30 m results in a seriously mixed pixel phenomenon that cannot exclude non-flooded roads and houses, increasing the uncertainty of flood recognition. Secondly, cloudy weather would lead to lower image quality interfering with flood extraction. Moreover, classifying paddy fields as flood areas also overestimated the flooded area.

### Extent and intensity of floods in three stages in 2020

**Figure 3:**
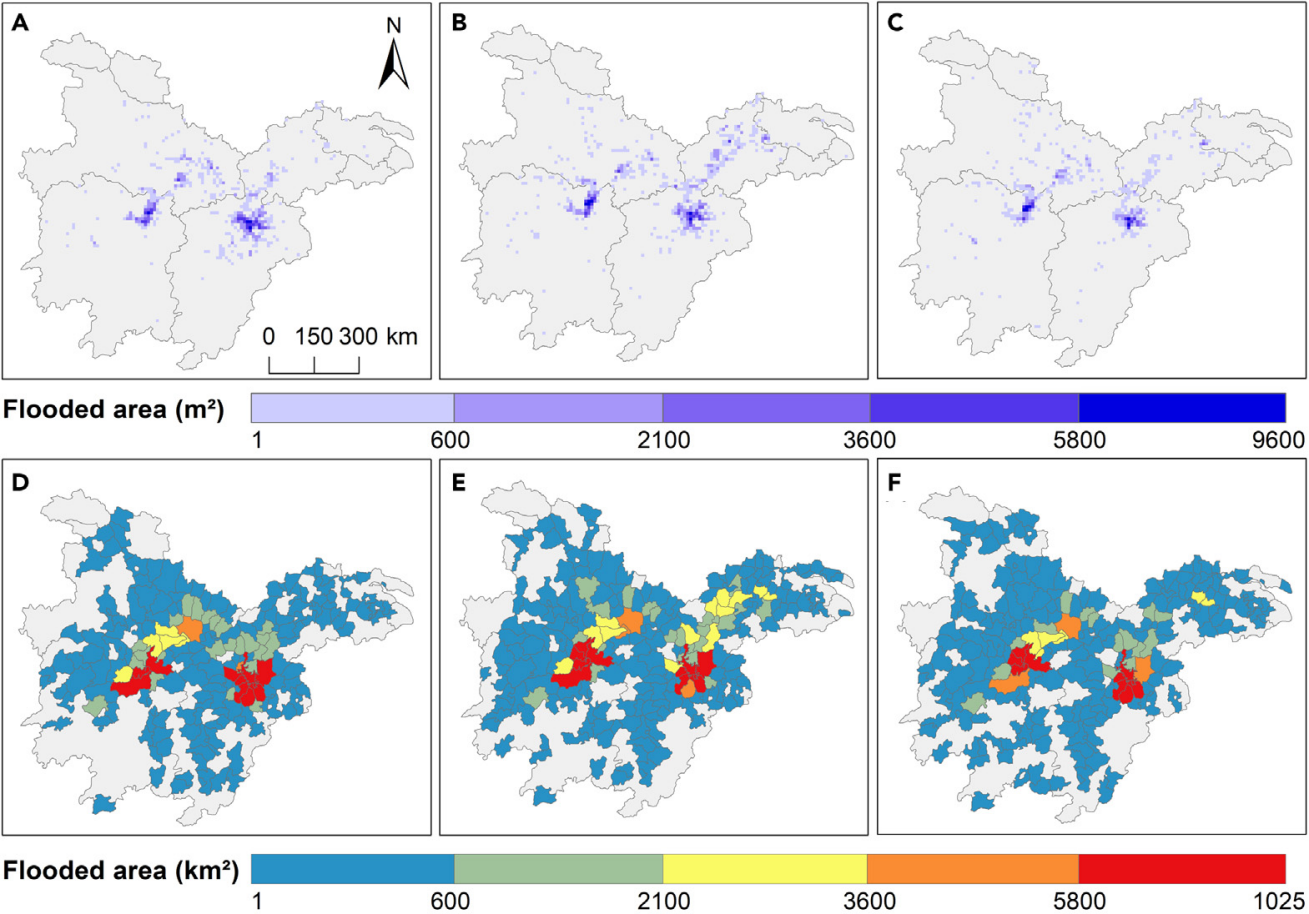
Flood extent in each stage (July. 1st to July. 22nd, July. 23rd to Aug. 13th, Aug. 14th to Aug. 31st) in 2020 (A–C) Flood extent of 10 km grid; (D–F) flood extent at the county level; (A, D) the stage of 2020.7.1–7.22; (B, E) the stage of 2020.7.23–8.13; (C, F) the stage of 2020.8.14–8.31.

The flood-affected area of the three stages (July. 1st to July. 22nd, July. 23rd to Aug. 13th, and Aug. 14th to Aug. 31st) was 3249 km^2^, 3652 km^2^, and 2450 km^2^ (Figures 3A–3C), respectively. Out of the 380 counties, floods affected 111, 133, and 124 counties (Figures 3D–3F) in the three stages. The second stage of flooding affected most counties, which was the flood control period that government departments had to pay the most attention to. The counties near the Dongting Lake Plain and Poyang Lake Plain were the most badly affected area, among which Jiujiang and Yugan County of Jiangxi Province were always in the top five flood-affected counties in the three-stage floods. Due to the severe shrinking of the lake area, especially Dongting Lake, the lake’s flood control capacity has been reduced.^35^ As soil erosion in the upper reaches of the river has produced a large amount of sediment silted up in the middle basin, the lake area has been shrinking year by year. The reclamation of the lake bank in the last century has dramatically damaged the lake’s function as a flood reservoir.^36^

**Figure 4:**
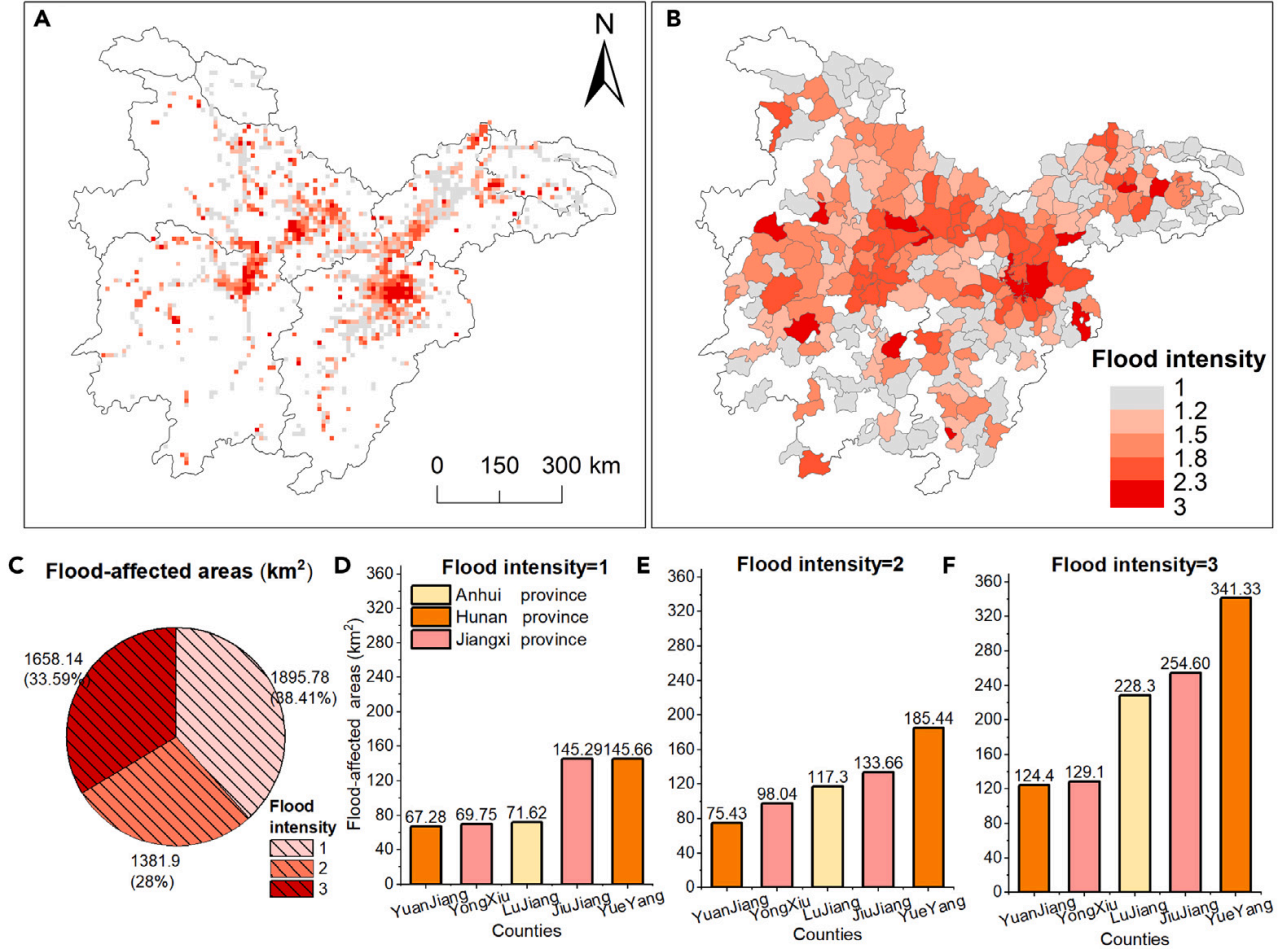
Flood intensity of 2020 (A) Flood intensity of 10 km grid; (B) flood intensity at the county level; (C) flood-affected area with different intensity. (D–F) The top five counties of the flood-affected area with different intensity.

In terms of flood intensity, areas of 1896 km^2^, 1382 km^2^, and 1658 km^2^ experienced floods once, twice, and thrice, respectively (Figure 4A), accounting for 38%, 34%, and 28% of the flood-affected area, correspondingly (Figure 4C). Figures 4A and 4B shows the spatial patterns of average flood intensity at 10 km and county scale. Large areas of high flood intensity were located around the lake or along the main channel due to rapidly rising water levels. Sporadic inland flooded areas suffered low flood intensity, possibly due to waterlogging caused by continuous rainfall during the rainy season. As for the flood-affected area with different intensities at the county scale (Figures 4D–4F), Yuanjiang County and Yueyang County in the Dongting Lake Plain and Poyang County in the Poyang Lake Plain were seriously affected by floods, which led to widely inundated area. Also, in all three counties, high-intensity areas (flood intensity = 3) were about twice the size of the low-intensity (flood intensity = 1) areas. It indicated that most flood-affected areas suffered repeated flooding that had constantly damaged agricultural land and artificial infrastructure. However, in low-lying areas near lakes or rivers, some severely inundated areas are flooded storage areas^37^ to prevent cities from flooding.

### Frequency of floods during 2015–2020

**Figure 5:**
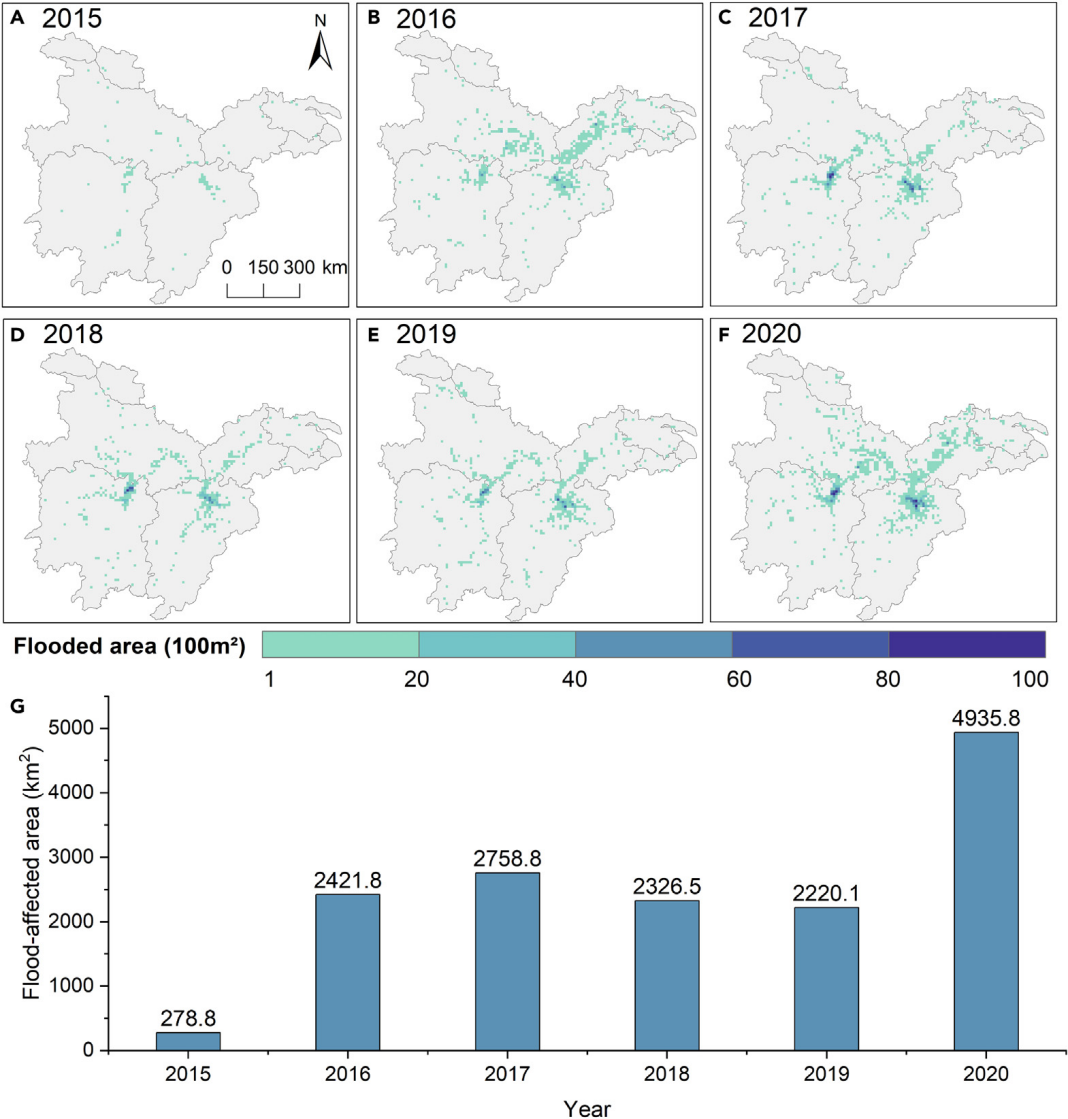
Annual flood extent of 2015–2020 (A–F) Flood extent of 10 km by 10 km grids in the form of flooded area percentages and interannual variations (G) of flooding in MLYP during the flood season.

During 2015–2020, the flood-affected area showed a remarkably increasing trend and reached a relatively high peak in 2017 (2759 km^2^) (Figure 5). In contrast, the 2020 flood was the most severe, with a flooded area of 4936 km^2^, almost twice the size of the other years.

**Figure 6:**
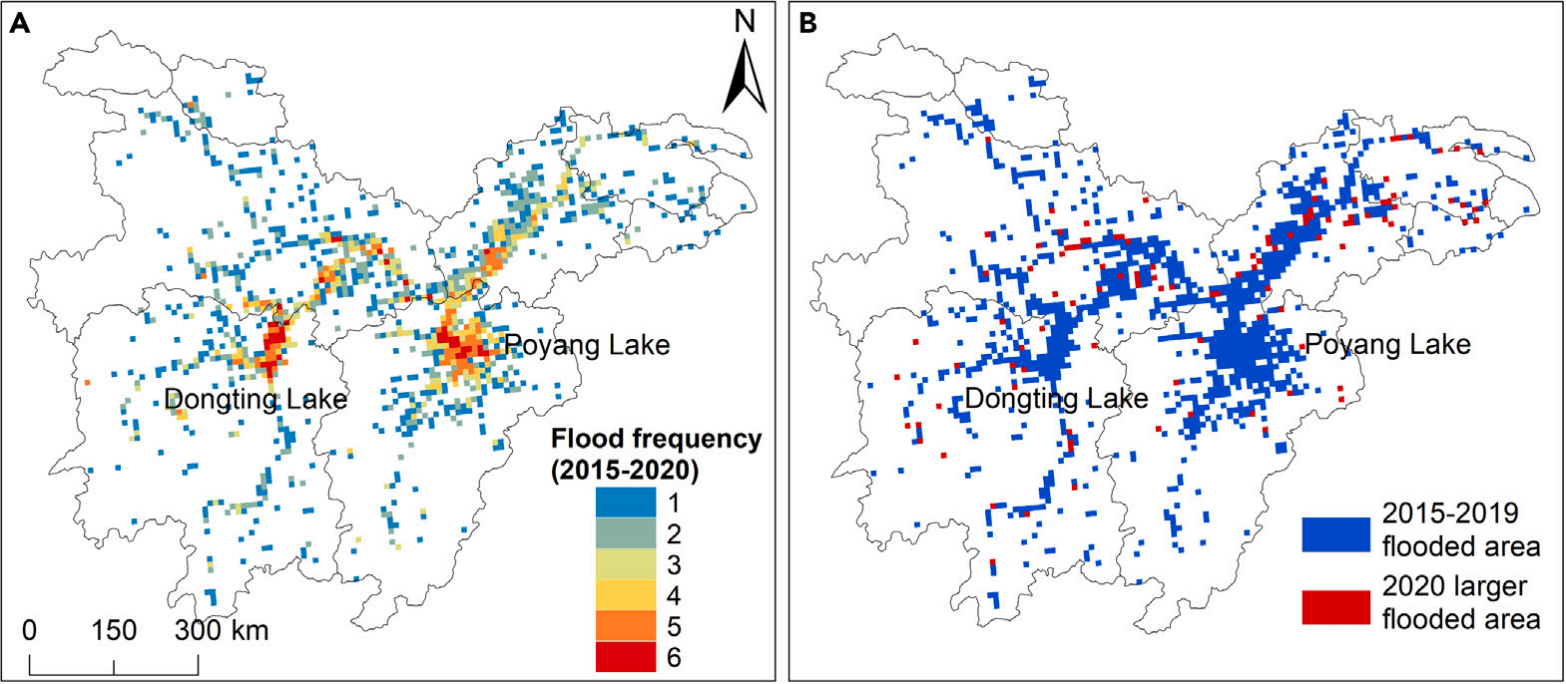
Severity of flooding in 2020 compared to previous years (A) S1-derived flood frequency (count) between 2015 and 2020. (B) Comparison with the maximum ever-flooded area in 2015–2019, the larger flood extent in 2020.

The frequently flooded areas were located near the lake or river (Figure 6A). Dongting Lake Plain and Poyang Lake Plain suffered flooding six times, representing every year of flooding in these areas in the 2015–2020 flood season. The Yangtze River and its tributaries were frequently flooded, three times near the main channel and mostly 1–2 times in the tributaries. The newly increased flood-affected region in 2020 (1671 km^2^) accounted for 29% of the maximum ever-flooded (5805 km^2^) from 2015 to 2019 (Figure 6B). It can be observed that the flooded area near the mainstream of the Yangtze River in 2020 was more extensive than in previous years.

### Assessment of the flood-affected croplands

**Figure 7:**
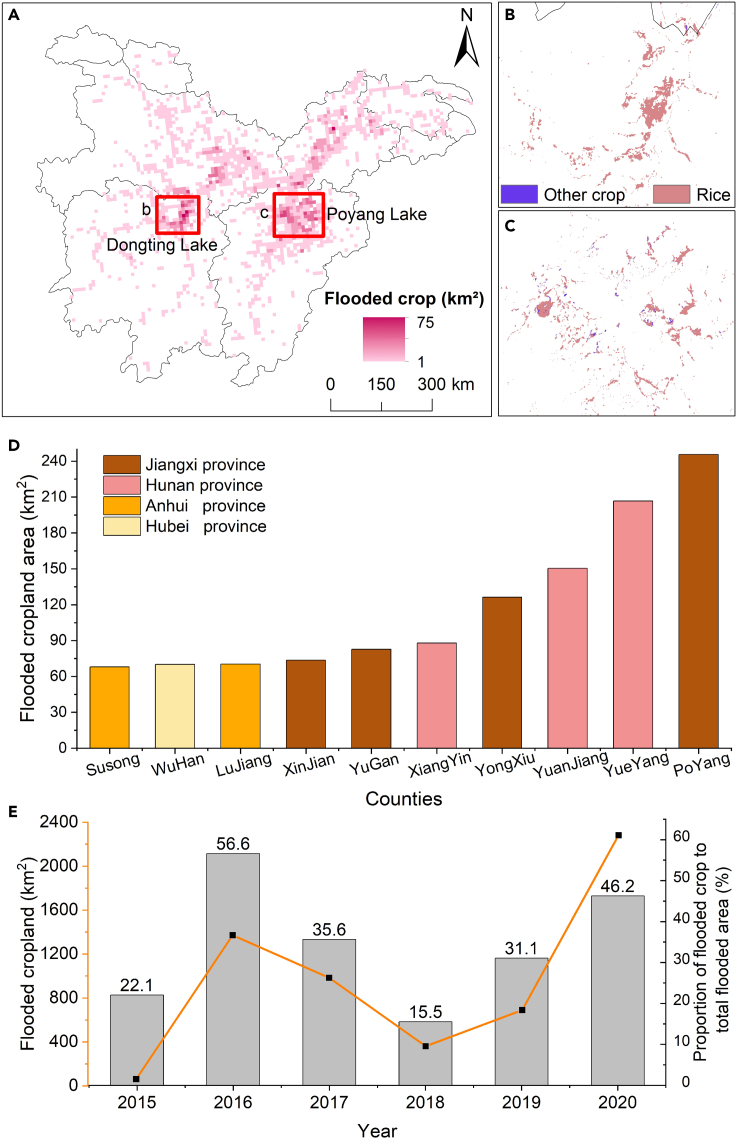
Flood-affected cropland fields in 2020 (A) 10 km grid area of cropland flooded by 2020; the flooded cropland area in zoom area of Dongting Lake Plain (B) and Poyang Lake Plain (C); (D) top 10 counties in the total number of flood-affected cropland area and (E) area of flooded croplands and the proportion of flooded area during 2015–2020.

The 2020 flood affected 2282 km^2^ of croplands and a total of 263 out of 380 counties (Figure 7A). The flooded croplands were mainly rice, accounting for 91%. Large areas of flooded cropland were located around Dongting Lake and Poyang Lake (Figures 7B–7C).

Located on the plains of the two lakes, Yueyang County in northeastern Hunan and Poyang County in northeastern Jiangxi were the largest flood-damaged cropland area compared with other counties (Figure 7D). Furthermore, 13% of croplands in Yuanjiang County were inundated, and the croplands in eastern Hubei and southern Anhui were also severely affected by floods. The 2020 flood affected the largest cropland area, with a value of 2282 km^2^, 46% of the total flooded area (Figure 7E). The flooded cropland area in 2016 ranked second, with a value of 1372 km^2^, 57% of the total flooded area.

**Figure 8:**
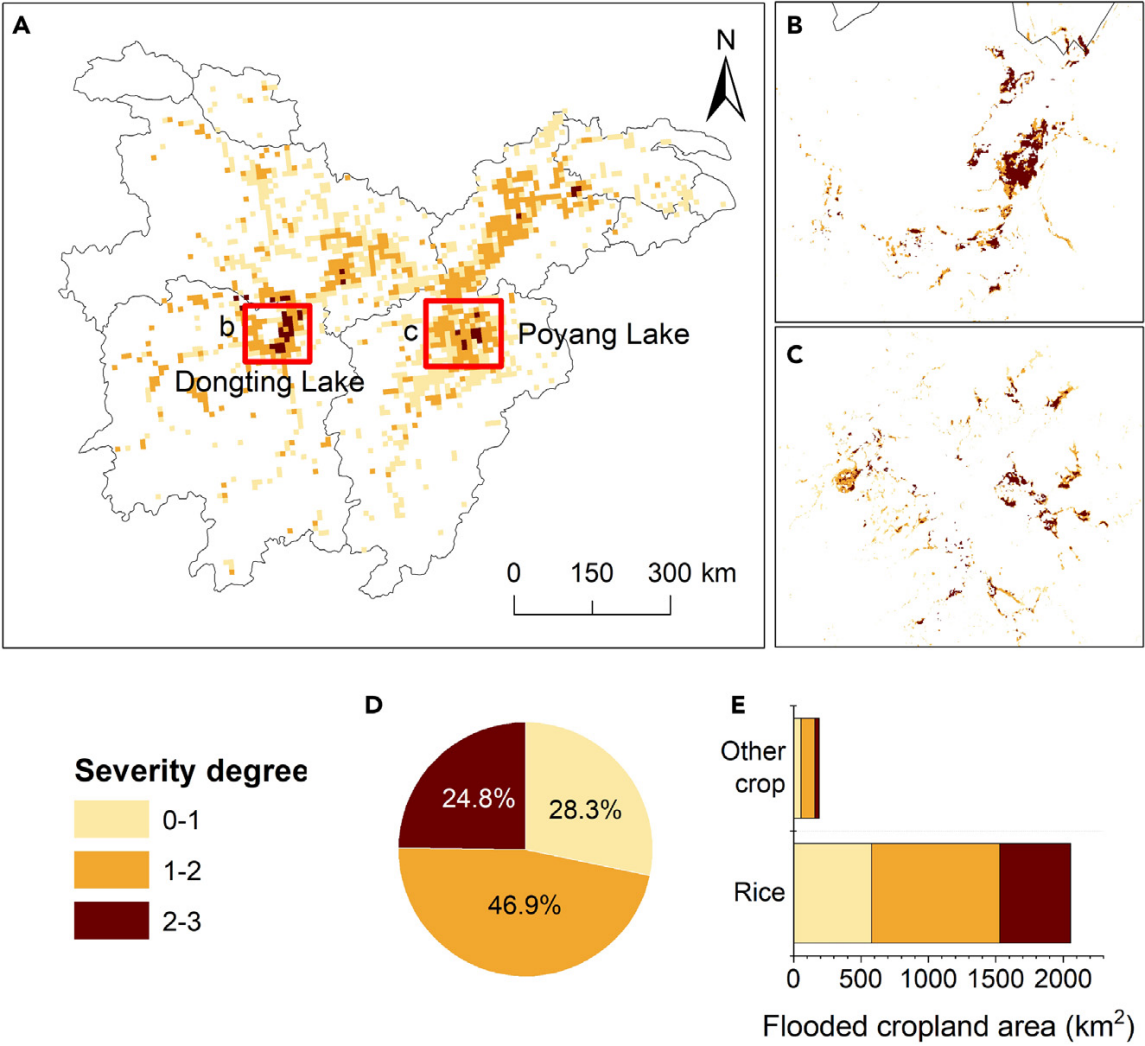
The severity degree of cropland damaged by floods (A) 10 km grid area of cropland severity degree; the damage cropland in zoom area of Dongting Lake Plain (B) and Poyang Lake Plain (C); flood-affected cropland area with different severity degrees (D) and different crop types (E).

Floods have caused different degrees of damage to cropland in different regions (Figure 8A). The severe damage regions (2 ＜ damage degree ≤ 3) were concentrated in Dongting Lake Plain (Figure 8B) and Poyang Lake Plain (Figure 8C), accounting for 25% (558 km^2^); while the moderate damage regions (1 ＜ damage degree ≤ 2) were mainly distributed in the mainstream plains of Yangtze River in Hubei and Anhui, accounting for 47% (1054 km^2^); and the slight damage regions (0 ＜ damage degree ≤ 1) were around the tributary plains of Hubei and Jiangxi province, accounting for 28% (637 km^2^). Nearly half of the cropland was moderately damaged, including 953 km^2^ of rice and 101 km^2^ of other crops (Figures 8D and 8E). The damage degree was calculated based on flood intensity and vegetation index reflecting vegetation growth. Such a distribution pattern implied that croplands suffered a low number of inundations and moderate damage to their growth. Farmers could compensate by replanting suitable crops such as vegetables as long as the water receded to reduce economic losses and avoid regional poverty.

## Discussion

This study proposed a framework for realizing the near real-time flood monitoring in MLYP during 2015–2020 based on integrating CDAT and NDFI algorithms, all available S1 imagery, and cloud computing in the GEE platform. Based on our flooded resulting maps, we comprehensively compared the floods in terms of the extent, intensity, and interannual variation. Moreover, the impact of floods on cropland was quantified by incorporating the resulting flood maps with the high-resolution cropland data, and the largest affected cropland was found in 2020. The accurate and timely description of flood severity could help execute flood prevention strategies to reduce economic loss and protect flood-prone cropland.

### Near real-time flood monitoring and comprehensive flood severity assessment

Compared to the existing studies of large-scale flood mapping mostly depicting the flood events with one map (DeVries et al. 2020; Di et al. 2018; Zhao et al. 2021a), we separated the flooding period into three time periods to analyze the dynamic pattern and inundated intensity, which supports the precise management and prevention in the severe flood-affected areas. Moreover, the interannual comparison reflects the inundated frequency and flood hotspots. Different from other studies that only provided flood frequency maps,^27^^,^^30^ our study analyzed the newly added inundated area of 2020 floods to measure the flood severity and future trends affecting new areas. These comprehensive results of high-resolution flood mapping in near real-time can be explained by the following facts.

(1) The observation of frequent flooding requires high temporal resolution remote sensing data.^38^^,^^39^ Previous studies often used high spatial resolution satellite images in monthly or even daily flood mapping at a small-scale.^33^^,^^34^ In addition, daily MODIS data could satisfy the frequent large-scale observation of floods; however, the spatially explicit flood map cannot be generated by its coarse resolution.^40^^,^^41^ In this study, we realized the large-scale flood mapping using all available S1 SAR data with 10m spatial resolution hosted in the GEE platform, which can ignore the weather effects and realize the near real-time flood monitoring by taking full advantage of a huge amount of data (Figure S3). Based on them, three large-scale flood maps for three flooding periods were obtained to enumerate the big flooding events during the summer of 2020 (Figure 3).
(2) Cloud computing platforms provide reliable support for large-scale flood monitoring promptly. Although remote sensing has unique advantages in flood monitoring, with effectiveness and large-scale coverage, compared to the traditional survey methods based on river gauge data and model simulations,^42^ it still has a significant demand for human and time resources. The GEE platform solves the difficulties of data acquisition and preprocessing, allowing us to monitor the floods in the entire MLYP effectively.
(3) The SAR-based algorithms are efficient and suitable for rapid flood mapping,^43^ whose performance depends on adequately understanding the differences in backscatter between flooded and non-flooded areas.^44^ However, it is difficult to completely distinguish the backscattering heterogeneity of dry and submerged vegetation using a single SAR image.^15^ In our study, we used two change detection algorithms (i.e., CDAT and NDFI) to analyze the multi-temporal data, which are easily implemented and permit quickly and automatically generate the large-scale flood maps, as well as the regional mobility with minimal change.

### Uncertainty analysis in flood mapping

There are some uncertainties in the process of flood mapping:

(1) The selection of the reference period. The reference period indicating the relatively dry or non-flooded scenarios was used to extract the additional flood extent, which is of great significance in flood mapping.^21^ Previous studies used the driest period to remove the influence of other floods,^21^^,^^27^ but falling river levels during the dry season could lead to overestimates of flood areas.^45^ Ideally, the reference period should be closest to the flood period, which could reduce the impact of seasonal changes in river and lake water levels, and ensure the image coverage for the study region.^45^ This study considered the relatively dry period closest to the flooding as the reference period to minimize the impact of seasonal changes in lake and water levels on the results;
(2) the setting of threshold values. One of the key issues in flood mapping based on a change detection algorithm is the threshold setting, and inappropriate thresholds may lead to the overestimation or underestimation of the flood extent. In addition to the previous studies^18^^,^^21^^,^^27^^,^^45^ that used the coefficient (Kc) value as 1.5 (Equation 3), we obtained the appropriate threshold (CDAT, −3.242 and NDFI, −0.244) based on the sample experienced analysis to better scale the change detection algorithm to this study area;
(3) the capture of the flood period. Although we used all available S1 imagery, the temporary flood area possibly had no satellite monitoring data (Figure S3). The revisit cycle of 6 or 12 days of S1 makes it challenging to obtain high-quality and large-scale image coverage in a short time interval, which cannot meet the requirements of near real-time monitoring of each flood event. Therefore, the five flood events in 2020 were combined into three stages synthesized by 22 days (July. 1st to July. 22nd, July. 23rd to Aug. 13th) and 18 days (Aug. 14th to Aug. 31st). As a result, the flood extent in the 2020 summer flood map might be smaller than that of the actual inundation area. In this case, harmonizing multi-sensor satellite images would help reduce the issues of missing ephemeral floods and provide more possibilities for near real-time flood mapping.^46^

### The impact of floods on croplands in the MLYP

Due to subtropical monsoons and typhoons, the plain area in the MLYP is one of the most rainstorm-prone areas in China.^47^ With the curved river channel and poor drainage, water flow is sandwiched between the hills, resulting in large flooded areas in the MLYP.^48^ Our study indicates that the flood-prone area during 2015–2020 is mainly located in the low-lying areas along the mainstream of the Yangtze River from Jingzhou County in Hubei to Tongling County in Anhui, especially in the Dongting Lake Plain and Poyang Lake Plain (Figure 6). As the biggest marketable grain base and important grain-cultivating areas in China,^49^ the MLYP is under enormous pressure in terms of food security.

Determining the impact of flood disasters on croplands with high-resolution cropland data can indicate detailed information on inundation and crop flood resistance,^50^ especially in the fragmented cropland in the MLYP. In this study, we overlayed the 10 m resolution cropland data^51^^,^^52^ with a 10 m flood map to assess the agricultural disaster caused by the floods during 2015–2020, especially the impact of the 2020 summer flood on food security. Although the flood-affected cropland in 2020 had the largest area (Figure 7), the proportion of the flooded area decreased compared with that in 2016. This might imply that significant progress has been made in constructing the agricultural disaster prevention and mitigation system in recent years. In the 13th five-year plan (2016–2020), the overall investment in water projects (including flood control) exceeded three trillion yuan (US$ 536 bn), according to the Ministry of Water Resources of the People’s Republic of China. In the MLYP, 46 flood storage and detention areas have been set up, with a total area of 12,000 km^2^ and an effective flood storage capacity of 59 billion m^3 53^. Previous studies^53^^,^^54^ indicate that using multiple retention basins could mitigate the flood risk for residential buildings and croplands in the downstream communities.

**Figure 9:**
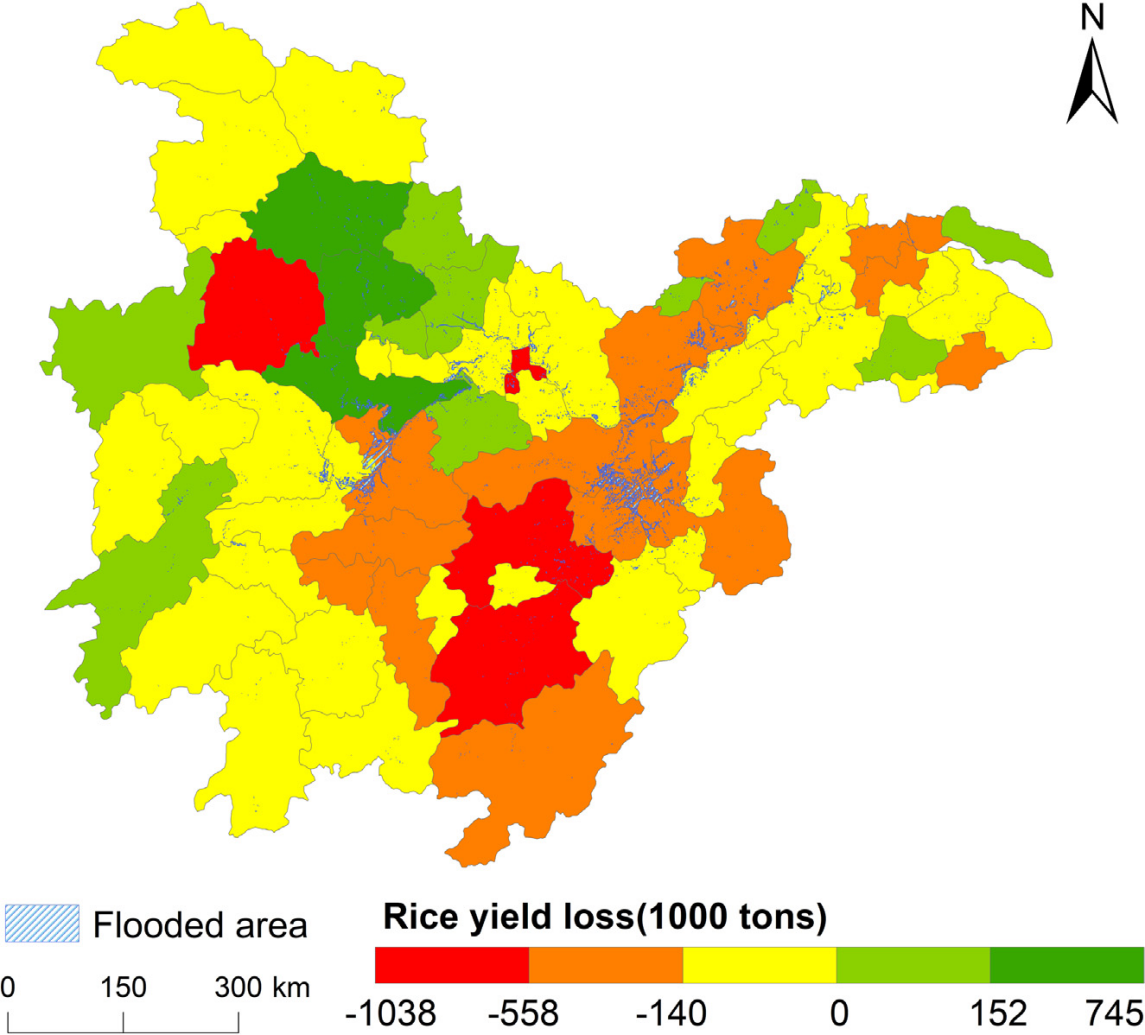
Rice production loss in 2020 at the city level compared to the average production in 2015–2019

Nevertheless, with the increase in extreme weather and climate events, it still faces challenges in agricultural disaster prevention. For example, only 28% of cropland suffered slight damage, and more than 1,500 km^2^ of rice fields suffered moderate or severe damage. Comparing the 2020 municipal rice production data provided by the local governments with the average production of previous years (2015–2019) decreased in 54 out of 67 cities with an average decrease of 140,000 tons that Jiangxi, Hunan, and Hubei all had more significant losses (Figure 9). The distribution pattern of rice damage is consistent with the affected croplands derived in this study. Therefore, large-scale, high spatial, and temporal resolution flood maps and crop maps with crop type and crop production details would be helpful for more precise damage assessments of flood-affected croplands.

### Potential implications of near real-time flood monitoring

The 2020 summer flood has recorded rainfall and flooding since 1998, which lasted up to two months, and several tributaries have exceeded historical maximum water levels,^48^ according to the MEM of the People’s Republic of China. Taking this extreme flood as a typical case, our results indicate that near-real-time flood monitoring is feasible in the large-scale region. Considering the flood intensity and interannual comparison, the wall-to-wall flood map would help the government to improve flood prevention projects and make proper post-flood arrangements in the perennial and newly added flooded areas to minimize casualties and property loss.^55^^,^^56^ Dongting Lake Plain and Poyang Lake Plain have suffered severe flooding, and local governments need to promote the implementation of the Return Farmland to Lake Program to restore lake flood storage capacity.^57^ In addition, perennially flooded areas should be closed to human settlement and regular flood management should be established in other flood-prone areas during the rainy season. Depending on the intensity of flooding, the governments could make appropriate and timely arrangements for the relocation of affected residents and property. For newly added flooded areas, the government needs to improve flood monitoring systems, as these floods have the potential to be catastrophic and may require greater attention to disaster response and rehabilitation plans.

Moreover, by combing with the high-resolution crop map and natural conditions (e.g., slope, topography, and soil), agricultural departments could use precision agriculture technology to schedule appropriate cropping patterns and restore timely agricultural production.^34^^,^^58^ For perennial flood plains where croplands were severely damaged, farmers should apply regenerative rice techniques or replace sowing double-season rice with early single rice, then replant vegetables or dryland crops after the flood season. As for areas with lower flood intensity and slightly damaged croplands, farmers should conduct rush harvesting before the flood season to minimize economic loss.

### Limitations of the study

This study developed a comprehensive framework of flood mapping and analysis of its spatial extent and intensity in near real-time and evaluated the 2020 flood-affected cropland area. Current limitations of the study are some uncertainties in the change detection algorithm that affect the accuracy of flood mapping (Detail in the section of uncertainty analysis in flood mapping), and the use of vegetation indices to reflect the degree of cropland damage is much less intuitive than cropland production.

### Conclusions

This study developed a near real-time method of large-scale flood monitoring based on all available S1 SAR data, two change-detection-based algorithms (i.e., CDAT and NDFI), and the GEE platform. Based on the proposed method, the high-precision maps of the summer floods in MLYP during 2015–2020 were produced. The results showed that the extent and intensity of the 2020 summer flood and its impact on the croplands were the largest in the previous six years. The flood-affected areas in 2020 were mainly concentrated in the Dongting Lake Plain and Poyang Lake Plain, and the Yangtze River mainstream, with inundated areas more than twice over areas in previous years, and newly added flooded areas accounted for 29% of the maximum flood-affected areas of previous years. In addition, more than 2200 km^2^ of croplands in MLYP were inundated in 2020 with more than half severely damaged. This study proposed a comprehensive framework of flood mapping and analyzed its spatial extent and intensity in near real-time, which could provide local governments with accurate and timely information for decision-making in reconciling flood prevention and agricultural development.

## STAR★Methods

### Key resources table

**Table:** 

REAGENT or RESOURCE	SOURCE	IDENTIFIER
**Deposited data**		

Raw and analyzed data	This paper	https://doi.org/10.17632/yz6mvby9rn.1
Sentinel-1 data	Google Earth Engine	https://developers.google.com/earth-engine/datasets/catalog/COPERNICUS_S1_GRD
Sentinel-2 data	Google Earth Engine	https://developers.google.com/earth-engine/datasets/catalog/COPERNICUS_S2_SR
Landsat-8 data	Google Earth Engine	https://developers.google.com/earth-engine/datasets/catalog/LANDSAT_LC08_C02_T1_L2
MOD13Q1	Google Earth Engine	https://developers.google.com/earth-engine/datasets/catalog/MODIS_061_MOD13Q1
MYD13Q1	Google Earth Engine	https://developers.google.com/earth-engine/datasets/catalog/MODIS_061_MYD13Q1
Cropland data	Cheng et al.^51^	N/A
Rice data	Han et al.^52^	http://www.nesdc.org.cn/sdo/detail?id=62c8f46b7e281714dccbd210
Rice production data	National Bureau of Statistics	https://data.cnki.net/yearBook?type=type&code=A
The digital elevation model (DEM) data of the Shuttle Radar Topography Mission (SRTM)	Google Earth Engine	https://developers.google.com/earth-engine/datasets/catalog/USGS_SRTMGL1_003
Precipitation data	CHIRPS dataset	https://www.chc.ucsb.edu/data/
Flood disaster events	EM-DAT data	https://www.emdat.be/
Flood disaster area in recent ten years	National Bureau of Statistics (NBS)	https://data.stats.gov.cn/

**Software and algorithms**		

Google Earth Engine	Google	https://earthengine.google.com/
ArcGIS10.2	Esri	https://desktop.arcgis.com/zh-cn/desktop/
MATLAB R2016a	Mathworks	https://www.mathworks.com/products/matlab.html
Synergic flood mapping by combining the CDAT and NDFI algorithms	This paper	https://code.earthengine.google.com/208610db2a1c940e732ce13371cfc81a

### Resource availability

#### Lead contact

Further information and requests for resources should be directed to the lead contact, Jinwei Dong, dongjw@igsnrr.ac.cn.

#### Materials availability

The flood maps generated in this study have been deposited on Mendeley at https://doi.org/10.17632/yz6mvby9rn.1.

### Method detail

The methodology of this study includes three steps: (1) Flood mapping. The preliminary flood maps were obtained using S1 SAR data and two change detection algorithms, CDAT and NDFI. After threshold setting and overlapping the two flood maps, the consistency map was generated as the actual flood map. (2) Accuracy assessment. Our flood maps were evaluated by sample points and S2 flood mapping. (3) Flooding impact analysis. The severity of summer floods in 2020 was evaluated through the three-stage flood maps in 2020 and the flood frequency map during 2015–2020; The flood-affected cropland was identified by overlapping cropland and flood maps and estimated the damage degree by the weighted sum method. Detailed descriptions of the methodology are provided in the following sections.

#### Study area and study year

The Yangtze River is the largest river in China and the third-largest river globally, which plays a vital role in China’s economic and social development.^59^ It is often attacked by floods in history, causing numerous civilian casualties and huge property losses.^60^ We selected the MLYP as the study area, which is the severely flooded-affected region of the Yangtze River basin.^61^ The MLYP (24°-34°N, 108°-121°E) locates in southern China, including Hubei, Hunan, Jiangxi, Shanghai, southern Anhui, southern Jiangsu, as well as the small parts of Shaanxi, Henan, and Zhejiang (Figure S1A). The annual precipitation ranges from 1000 mm to 1500 mm, with frequent rainstorms in summer and autumn.^62^ The majority of precipitation (59–89%) occurs from June to August, when flooding events often occur (Figure S1B), with over 2000 km^2^ of flood-affected areas during 2011–2020 (Figure S1C).

In 2020, due to the combined effects of the subtropical high in the western Pacific, the westerly zone, snow cover on the Qinghai–Tibet plateau, and global climate anomalies (El Niño),^48^ the continuous heavy precipitation primarily occurred in July and August in the Yangtze River basin (Figure S1D). In addition, the MLYP is one of the national agricultural production bases in China.^63^ The frequent flood events give rise to waterlogging disasters in low-lying farmland, affecting the growth and development of croplands, such as corn, cotton, vegetables, and economic fruits. Summer floods mainly affected double-cropped early rice at harvest time, late rice at planting time, and single-cropped rice at late tillering or booting stage.^34^^,^^64^ The total grain production and added value of the agricultural sector in 2019 accounted for 35.9% and 43.4% of the total production in China.^65^ The MLYP has become the biggest marketable grain base in China, with a high commodity rate of over 40%.^49^

According to the severity of the flood, the Ministry of Water Resources (MWR) of the People’s Republic of China marks annual floods as numbered floods.^66^ Exceeding the warning water level, the magnitude of two to five years flood or affecting the safety of local flood control are taken as flood numbering criteria.^66^ In 2020, five severe floods were numbered in the Yangtze River basin (Table S1). Based on the duration of the five floods, we considered the period from July 1 to August 31 as the flood period of the MLYP, which is further divided into three stages (Jul. 1 to Jul. 22, Jul. 23 to Aug. 13, Aug. 14 to Aug. 31).

#### Data and preprocessing

##### Satellite data

In this study, we used Sentinel-1 (S1) data during 2015–2020 for flooding detection. The Sentinel-1 is a Synthetic Aperture Radar (SAR) that collects data all-weather, day or night, with an active microwave sensing technique. The sensor carries a C-band (4–8 GHz) SAR instrument that supports four exclusive imaging modes providing different resolutions and coverages: Interferometric Wide Swath (IW), Extra Wide Swath (EW), Strip Map (SM), and Wave (WV). We used the IW mode, which has been proven to be more suitable for flood detection due to higher data availability.^27^^,^^67^ The IW-mode SAR imagery is provided in dual-polarization with vertical transmit and vertical receive (VV), and vertical transmit and horizontal receive (VH). Previous studies have demonstrated that the VV polarization data are more suitable for accurate flood detection than the VH data.^68^ The spatial resolution of the data is 10 m, with a 6 or 12 days revisit cycle.^18^^,^^45^ We acquired the calibrated, ortho-corrected S1 data from Google Earth Engine (GEE), where each S1 scene has been preprocessed with S1 Toolbox: (1) thermal noise removal; (2) radiometric calibration; (3) terrain correction using SRTM 30 or ASTER DEM for the northern areas with latitudes higher than 60 degrees. The final terrain-corrected values are converted to decibels via log scaling (10×log10(x)). Due to the change of radar return in pixels caused by vegetation and other scattering sources, SAR suffers from speckle which leads to random changes in the pixel’s brightness and usually hampers decision-making on a pixel basis.^69^^,^^70^ Filters were applied to remove speckles, and smoother images were achieved, which can be more accurately used in further processing.

The Sentinel-2 (S2) data in 2020 was also used to extract the water body for cross-comparison. S2 is a wide-band, high-resolution, multi-spectral remote sensing data. In this study, we used the visible and near-infrared bands with a 10-m spatial resolution and the 20-m short-wave infrared band to calculate the water and vegetation indices.

In addition, we acquired the 250-m MODIS Vegetation Indices product (MOD13Q1 and MYD13Q1) with a 16-day temporal resolution from 2015 to 2020. The MODIS products provide two primary vegetation layers, the Normalized Difference Vegetation Index (NDVI) and Enhanced Vegetation Index (EVI), which were used for cropland damage assessment.

All the above data, including Landsat-8 (L8) data for 2020, were used to collect training and validation data, which were archived on the GEE platform as image collection. The Landsat-8 Operational Land Imager (OLI) from the United States Geological Survey (USGS) has 30-m spatial resolution and 16-day temporal resolution.

##### Cropland data

To study the impact of flood disasters on croplands, we integrated two cropland products to delineate the extent of the cropland. The products included 10-m cropland data in 2015 derived from the First National Geography Census^51^ and a 500-m annual paddy rice map from 2015 to 2020.^52^ The 10-m cropland data was generated using visual interpretation and field survey methods with an accuracy higher than 99.7%. The rice map was generated by the improved phenology-based methods based on MODIS products with R2 ranging from 0.85–0.95. We obtained the maximum extent of rice planting area from 2015 to 2020 from the rice map. Owing to the summer floods mainly affecting rice, the cropland data for flood impact analysis were generated by integrating the existing cropland data and resampled 10-m spatial resolution rice map.

##### Other data

The digital elevation model (DEM) data of the Shuttle Radar Topography Mission (SRTM)^71^ with the 30-m spatial resolution was also used to exclude non-flooded hilly terrain pixels. The precipitation data from the CHIRPS dataset (https://www.chc.ucsb.edu/data/),^72^ the flood disaster events from the EM-DAT data (https://www.emdat.be/)^73^ and the flood disaster area from the National Bureau of Statistics (NBS) (https://data.stats.gov.cn/) in recent ten years were used for the selection of flood periods and reference period in the study.

##### Training and validation data

We randomly collected training and validation samples to evaluate the flood maps from high/medium resolution satellite images, including 10-m S2 images, 10-m S1 images, 30-m Landsat 8 images, and 500-m MODIS data. Samples with mixed water/land pixels were rejected to ensure pixel purity. Then, we manually verified each remaining sampled pixel and labeled them as "flooded" or "non-flooded" based on the above-mentioned multi-source datasets. A total of 3000 samples were collected, of which flooded and non-flooded sample numbers were 1290 and 1710, respectively. The distribution of samples is shown in Figure S2.

##### Flood mapping

###### The change detection algorithms

(1) The Change Detection and Thresholding (CDAT) algorithm. The CDAT algorithm is an adapted change detection and thresholding method using multi-temporal S1 images to map floods.^21^ The following steps were applied: (a) reference image and flood image acquisition: create two S1 multi-temporal series, one containing reference images (R, The detail of reference period selection in section of selection of appropriate reference period) and one containing flood inundation images (F). We calculated the minimum VV for both stack images. (b) change detection by band math: use the reference image and flood image to generate a difference image (D) (Equation 1); (c) thresholding setting: extract flooded areas using decision tree classification (Detail in the section of filter and threshold setting).

(Equation 1) D=min⁡(F)−min⁡(R)

(2) The Normalized Difference Flood Index (NDFI) algorithm. The NDFI algorithm highlights flooded areas from normal surface conditions and temporarily covered water 18. The average backscattering values in the multi-temporal reference image stack represent the normal features of the surface, including low values from smooth surfaces and high values from rough surfaces. Combining the minimum values in the reference (R) and flood image stacks (F) captures extremely low backscatter values caused by flooding. The difference between the average and minimum values highlights the low backscatter value, i.e., the flooded area. The NDFI was calculated as shown:

(Equation 2) $$NDFI=\frac{mean\left(R\right)-\min\left(R+F\right)}{mean\left(R\right)+\min\left(R+F\right)}$$

(3) Synergic flood mapping by combining the CDAT and NDFI algorithms. Overlapping the flood maps generated using CDAT and NDFI algorithm, the consistency map was obtained from the common flooded area. As the actual flooding, this consistency map could reduce the uncertainty caused by speckles^27^ and be used for further analysis.^74^ The annually flooded areas were stacked to show the flood frequency and reflect the extent and intensity of floods in 2020.

###### Selection of appropriate reference period

Ideally, The two periods of non-flood reference and flood inundation should be on the same dates in different years^45^ to eliminate errors due to water area differences caused by seasonal changes in lakes and rivers. However, as the MLYP suffers from floods in July and August every year, the similar period of previous years is unsuitable for reference. Considering the local climate,^18^ the appropriate reference period should be near the rainy season and relatively dry. Precipitation in the MLYP has increased since May (Figure S1B), with correspondingly increasing flood events that last until late August. So, we chose March to April as the reference period.

Due to the relatively long revisit period of S1, another challenge in selecting the reference period is ensuring each satellite track’s image coverage in the MLYP. The valid observations in March-April 2015 were relatively sparse (Figure S3). So, we adjusted the reference to another non-rainy season, September-December. More than one observation in each pixel indicates that the S1 images could support the annual flood mapping in the MLYP.

###### Filter and threshold setting

The threshold setting used the decision tree to classify and exclude non-flooded pixels in the preliminary coarse flood maps generated by CDAT and NDFI. The procedure was conducted through three steps:

(1) Terrain filter. Exclude non-flooded hilly terrain pixels. The decision tree starts with a masking decision that determines whether the pixel of the difference image has a slope less than five degrees (≤5°) by analyzing the input DEM. Masking these steeper slopes, such as river banks, hydraulic structures, and hill slopes, removed brightness changes in pixels caused by the signal angle returned from the hillside.^75^
(2) Background pixels filter. Exclude non-flooded background pixels. The pixels with a different image of zero were excluded.^21^
(3) Land cover seasonal change filter. Similar to the flood, the seasonal change of land cover occasionally reduces backscatter in the different images.^18^ The global threshold based on the histogram of the flood image could extract the land-water boundary. After comprehensively analyzing several flood images, we proved threshold of −19 was particularly effective.
(4) Threshold setting. After removing the non-flooded pixels, the threshold (T) was calculated as follows:

(Equation 3) $$T=mean\left[I\right]-K_{c}*std\left[I\right]$$

where *I* is the value of CDAT difference image or NDFI index. The optimum coefficient (K_c_) is 1.5,^18^^,^^21^^,^^27^^,^^45^ which might be fine-tuned for particular cases. We randomly selected the ground truth samples (Figure S2) which 1000 samples were for training and 2000 samples for validation. We repeated the process five times to determine the final threshold with the highest accuracy (Table S2). The threshold values of CDAT and NDFI were set to −3.242 (Figure S4A) and −0.244 (Figure S4B), respectively. Pixels with values below threshold values were attributed to flooded pixels.

##### Accuracy assessment

Firstly, we used the training and validation samples (Figure S2) to assess flood maps with four types of statistical indices, total accuracy (OA), user accuracy (UA), producer accuracy (PA), and F-score.

Secondly, we intercompared the S1-based flood map with the S2-based flood map.^76^ The S2 flood map was extracted using the dry and rainy season images, which could distinguish between permanent water and flooded areas. Although the S2-based flood map may not be accurate because of frequent clouds in the study area, it could evaluate the spatial pattern of the SAR-based flood map. We used three widely used spectral indices to extract the flooded areas (F)^77^: the Normalized Difference Vegetation Index (NDVI),^78^ Enhanced Vegetation Index (EVI),^79^ and the Land Surface Water Index (LSWI),^80^^,^^81^ which were defined as (Equation 4), (Equation 5), (Equation 6):

(Equation 4) $$NDVI=\frac{NIR-RED}{NIR+RED}$$

(Equation 5) $$EVI=\frac{2.5*\left(NIR-RED\right)}{NIR+6*RED-7.5*BLUE+10000}$$

(Equation 6) $$LSWI=\frac{NIR-SWIR1}{NIR+SWIR1}$$

(Equation 7) F=(LSWI>EVI)AND(NDVI>0.1)

where RED, BLUE, NIR, and SWIR1 are the reflectance of the red, blue, near-infrared, and short-wave-infrared bands of Sentinel-2 image.

##### Comprehensive assessment of floods

The comprehensive analysis of flood severity includes the extent and intensity in one year and multi-year frequency of floods from 2015–2020. To further explore the flood situation in the summer of 2020, flood inundations of three stages (Jul. 1 to Jul. 22, Jul. 23 to Aug. 13, and Aug. 14 to Aug. 31) were extracted. We scaled the flood map to 10 km × 10 km to depict the flood pattern and reflect the situation of flood-affected cities with the statistical area at the county level. In addition, the intensity of the 2020 floods was analyzed by overlaying the flood maps of the three stages to obtain the flood intensity map and counting areas of flood-affected areas with different intensities at pixel level and county level.

To assess the unprecedented severity of the catastrophic flood in 2020, we extracted flooded areas in all six years to generate the inundation frequency of the 10-km flood map. It could depict the spatial pattern of flood hotspot areas and the additional flood extent in 2020 compared with the maximum ever-flooded in 2015–2019.

##### Cropland damage assessment

This study combined flood information, crop types, and crop condition profile for Cropland damage assessment. By overlaying the high-precision cropland data with flood maps during 2015–2020, this study monitored the extent of affected cropland in the 2020 catastrophic flood. Moreover, we estimated the degree of cropland damage by weighing the sum of flood intensity and three vegetation indices, including NDVI, EVI, and disaster vegetation damage index (DVDI).^82^ The degree of cropland damage could be rapidly assessed by comparing vegetation indices before and after a flood event.^83^^,^^84^^,^^85^

We compared the difference in vegetation indices between the year of flood and the historical year for the same period (September-October) to reflect the impact of flooding, calculated as follows:

(Equation 8) $${NDVI}_{d}={NDVI}_{a}-{NDVI}_{b}$$

(Equation 9) $${EVI}_{d}={EVI}_{a}-{EVI}_{b}$$

where a and b are the mean value of the vegetation index in the period after and before flood, respectively. DVDI requires all NDVI values from 2000 to the year before the flood event as a historical series to calculate (Equation 10).

(Equation 10) $$\left\{ \begin{array}{c} mVCI=\frac{NDVI-{NDVI}_{med}}{{NDVI}_{\max}-{NDVI}_{med}}\\ DVDI={mVCI}_{a}-{mVCI}_{b} \end{array}\right.$$

where NDVI is the mean value of NDVI in the period; NDVI_med_ and NDVI_max_ are the median and maximum value of NDVI in historical time series, respectively; mVCI_a_ and mVCI_b_ are the vegetation conditions after (September-October) and before (June-July) flood respectively. The severity degree of cropland damage was calculated as follows:

(Equation 11) $$Severity\;degree=\frac{1}{4}\times \left({NDVI}_{reclass}+{EVI}_{reclass}+{DVDI}_{reclass}+intensity\right)$$

where NDVI_reclass_, EVI_reclass_, DVDI_reclass_ are the reclassed results of (Equation 5), (Equation 6), (Equation 7) that were divided into four categories using the geometrical interval method, with 0 representing no damage and 1–3 representing different levels of damage. Intensity is flood intensity, with values ranging from 1 to 3 representing the number of inundations in the 2020 summer three-stage floods.

## Data Availability

• This paper analyzes existing, publicly available data. These accession numbers for the datasets are listed in the key resources table. • All original code has been deposited on Google Earth Engine and is publicly available. Links are listed in the key resources table. • Any additional information required to reanalyze the data reported in this paper is available from the lead contact upon request.
