# Supplementary material for: Characterizing the 2020 summer floods in South China and effects on croplands
Source: iScience. 2023 Jun 12;26(7):107096. doi: 10.1016/j.isci.2023.107096 (PMC10319219; doi:10.1016/j.isci.2023.107096)
Supplement: Document S1. Figures S1–S4 and Tables S1 and S2 [file mmc1.pdf]

iScience, Volume *26*

Supplemental information

Characterizing the 2020 summer floods in South China and effects on croplands

Xi Chen, Jinwei Dong, Lin Huang, Lajiao Chen, Zhichao Li, Nanshan You, Mrinal Singha, and Fulu Tao

Supplemental information

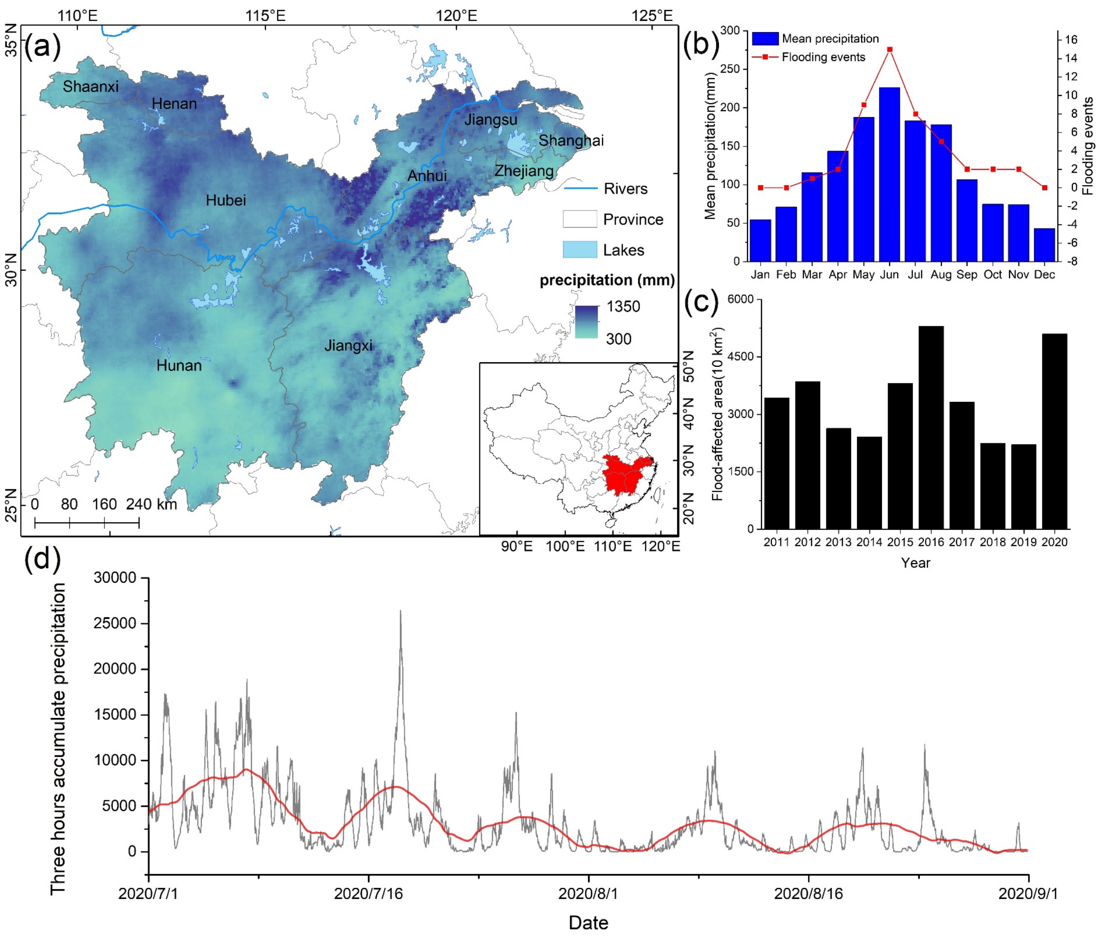

Figure. S1. Brief introduction of the middle and lower reaches of the Yangtze River Plain (MLYP), related to STAR Methods. (a) Location of the MLYP with the precipitation distribution from June 1, 2020, to August 31, 2020; (b) Seasonal variation of mean monthly rainfall during 2000-2020 based on the CHIRPS-v2.0 data [1] and flooding events during 2014-2020 derived from the EM-DAT data [2]; (c) Interannual variations of flood-affected areas caused by geological disasters and typhoons in the MLYP during 2011-2020, according to the National Bureau of Statistics of China (NBSC); (d) Mean accumulate precipitation in 2020 flood period in the MLYP derived from the IMERG-v06 data [3], with the red smooth curves reflecting the five floods in the summer of 2020.

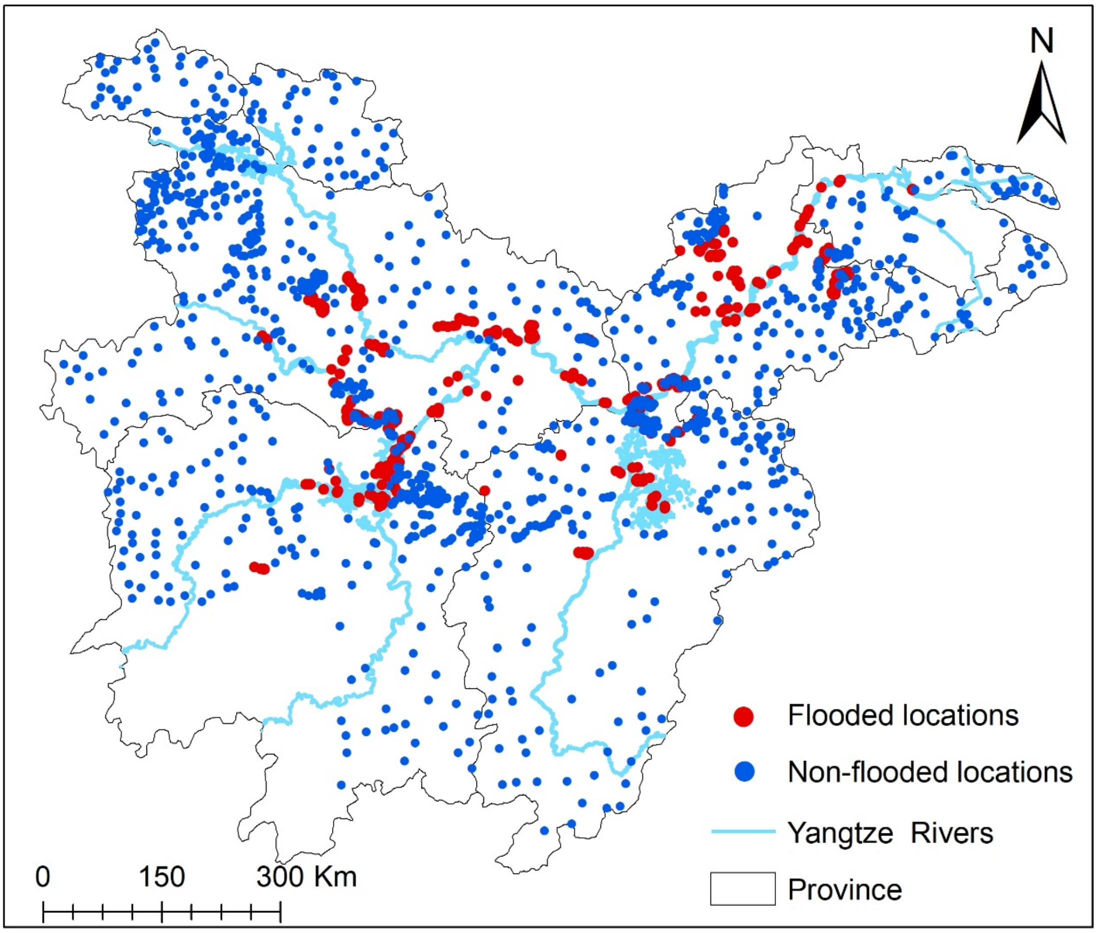

Figure. S2. Spatial distribution of the validation samples for accuracy assessment in flood mapping, related to STAR Methods.

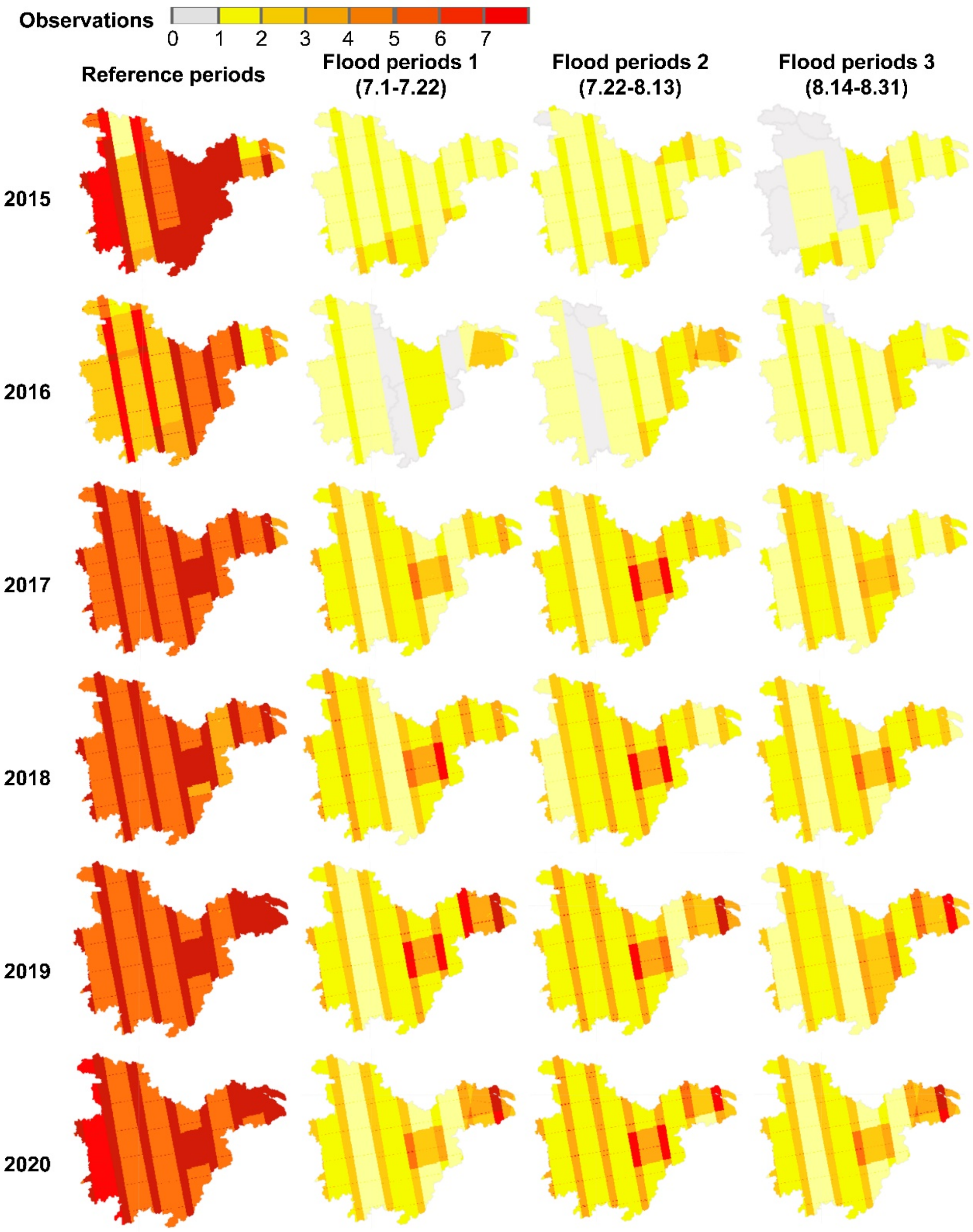

Figure. S3. Availability of time series S1 images during the study period (reference period (March to April) and flooded period (July to August) in 2015-2020, related to STAR Methods.

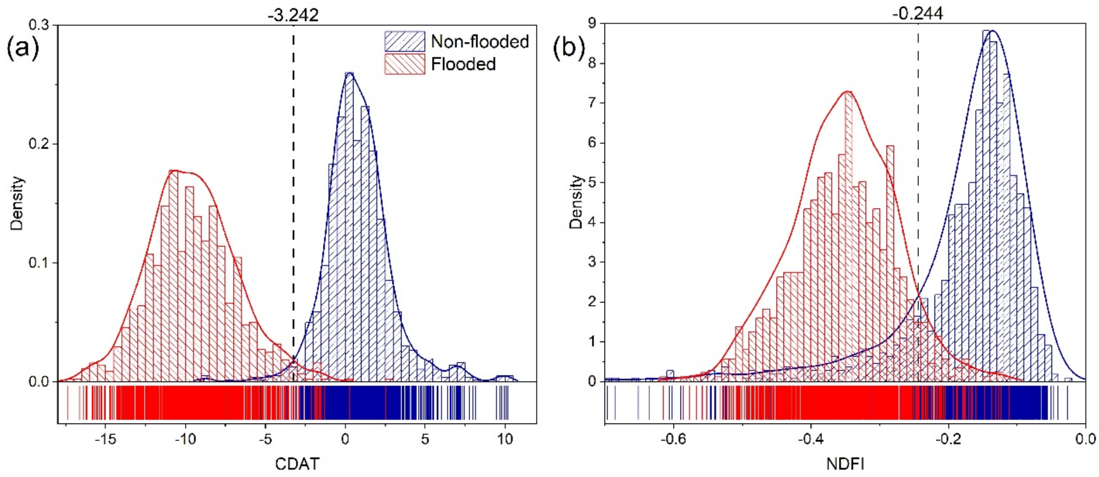

Figure. S4. The threshold setting of CDAT and NDFI, related to STAR Methods. Frequency distribution histogram of samples counted by CDAT (a) and NDFI (b).

Table S1. The five number floods in the Yangtze River basin in the summer of 2020, related to STAR Methods. The data is obtained from the Changjiang Water Resources Commission of the Ministry of Water Resources (CWRC) (http://www.cjw.gov.cn/).

Stages	Flood No.	Duration	Flooding situation
**Stage I**	No.1 flood	7.2-7.12	Precipitation was mainly concentrated in the middle and lower reaches, and the river level of mainstream exceeded the warning water level.
	No.2 flood	7.17-7.22	The lower reaches of Jianli in Hubei Province were all in a state of high alert, and the Three Gorges reservoir had ushered in a flooding process of 61000 m^3 / s.
**Stage II**	No.3 flood	7.26-8.13	Due to the heavy rainfall in the upper reaches of the Yangtze River, the water level rapidly raised.
**Stage III**	No.4 flood	8.14-8.17	It caused flooding in many areas of the Sichuan Basin, inundation of low-lying areas, some water sections in the MLYP exceeded the warning water level, and further increased flood control pressure in the lower reaches.
	No.5 flood	8.17-8.22	The mainstream of the Yangtze River had greatly exceeded its capacity of water level and the high-water level of the middle and lower mainstreams lasted long. The Three Gorges reservoir has ushered in the maximum discharge of 74000 m^3 / s since its construction.

Table S2. F1 value distribution for five different threshold settings, related to STAR Methods.

CDAT	NDFI	F1
**-3.242**	-0.244	0.956
**-3.363**	-0.238	0.948
**-3.214**	-0.245	0.928
**-3.278**	-0.245	0.943
**-3.245**	-0.245	0.935

References

1. Funk, C., Peterson, P., Landsfeld, M., Pedreros, D., Verdin, J., Shukla, S., Husak, G., Rowland, J., Harrison, L., and Hoell, A. (2015). The climate hazards infrared precipitation with stations—a new environmental record for monitoring extremes. Scientific data *2*, 1-21.
2. Guha-Sapir, D., Below, R., and Hoyois, P. (2016). EM-DAT: the CRED/OFDA international disaster database.
3. Tan, J., and Huffman, G.J. (2019). Computing Morphing Vectors for Version 06 IMERG. NASA/GSFC: Greenbelt, MD, USA.
